# Investigation of tension wood formation and 2,6-dichlorbenzonitrile application in short rotation coppice willow composition and enzymatic saccharification

**DOI:** 10.1186/1754-6834-4-13

**Published:** 2011-05-24

**Authors:** Nicholas JB Brereton, Frederic E Pitre, Michael J Ray, Angela Karp, Richard J Murphy

**Affiliations:** 1 Division of Biology, Imperial College London, South Kensington Campus, London SW7 2AZ, UK; 2 Plant and Invertebrate Ecology Department, Centre for Bioenergy and Climate Change, Rothamsted Research, Harpenden, Hertfordshire AL5 2JQ, UK; 3 Institut de recherche en biologie vegetale, University of Montreal, 4101 Sherbrooke East, Montreal, QC H1X 2B2, Canada

## Abstract

**Background:**

Short rotation coppice willow is a potential lignocellulosic feedstock in the United Kingdom and elsewhere; however, research on optimising willow specifically for bioethanol production has started developing only recently. We have used the feedstock *Salix viminalis *× *Salix schwerinii *cultivar 'Olof' in a three-month pot experiment with the aim of modifying cell wall composition and structure within the stem to the benefit of bioethanol production. Trees were treated for 26 or 43 days with tension wood induction and/or with an application of the cellulose synthesis inhibitor 2,6-dichlorobenzonitrile that is specific to secondary cell walls. Reaction wood (tension and opposite wood) was isolated from material that had received the 43-day tension wood induction treatment.

**Results:**

Glucan content, lignin content and enzymatically released glucose were assayed. All measured parameters were altered without loss of total stem biomass yield, indicating that enzymatic saccharification yield can be enhanced by both alterations to cell wall structure and alterations to absolute contents of either glucan or lignin.

**Conclusions:**

Final glucose yields can be improved by the induction of tension wood without a detrimental impact on biomass yield. The increase in glucan accessibility to cell wall degrading enzymes could help contribute to reducing the energy and environmental impacts of the lignocellulosic bioethanol production process.

## Background

In the production of bioethanol from lignocellulosic biofuel crops, one of the principal energy inputs arises from the need for a severe pretreatment of the cell wall matrix prior to enzymatic saccharification to increase access to the structural sugar polymers [1]. This recalcitrance property is believed to be due to several cell wall factors such as the lignin and hemicellulose content, their composition and structure as well as cellulose content, ultrastructure and degree of polymerisation [2]. The degree to which these elements affect recalcitrance, and therefore the energy balance of the whole lignocellulosic biofuel process chain, is currently the focus of much research and debate. Amongst the crop feedstocks available, there is considerable potential for short rotation coppice (SRC) willow to be used as a dedicated bioenergy crop for lignocellulosic biofuel production [3,4]. However, there has been little investigation or optimisation of the wood quality and composition of SRC willows for this end use.

Wood properties can be altered in response to environmental factors such as gravity and resource availability [5]. Tension wood formation is a natural response in angiosperms to reorient stem growth towards the vertical. This tension wood is characterised by gelatinous fibres (G fibres) that develop exclusively on the 'upper' side of the responding stem. G fibres contain a unique cell wall layer internal to the secondary cell wall, termed the 'gelatinous layer' (G layer). The G layer is composed almost entirely of cellulose (88.6%) in *Populus alba*, with some evidence indicating xyloglucan as the major noncellulosic constituent [6]. However, little work has been performed to measure the chemical composition of 'opposite' wood, formed on the opposite (lower) side of tension wood in the reaction wood stems of angiosperms. Some previous work in conifers has provided evidence that opposite wood (to the gymnosperm compression wood) has the same chemical composition as normal wood [7].

Two major methods have been used for the experimental induction of tension wood: bending, with the most extreme induction being a loop of the stem, and inclining of the stem with restraint using an immobile support [8,9]. The degree of induction by inclination at several angles has been tested, with 120° found to elicit the greatest amount of tension wood [10].

The compound 2,6-dichlorbenzonitrile (DCB) is a cellulose synthesis inhibitor commercially used as a preemergence herbicide, but it has also been used as a tool for investigating cell wall assembly and stress [11-15]. In *Arabidopsis thaliana*, DCB treatment of the cell wall has been shown to result in membrane/cell wall adhesion site hyperaccumulation of *AtCESA-6*, a cellulose synthesis subunit whose expression is specifically upregulated during secondary cell wall synthesis. The treatment inhibited mobility, resulting in dwarf phenotypes [12]. DCB specifically binds to a microtubule-associated protein, PttMAP-20, whose expression is also normally upregulated during secondary cell wall synthesis in poplar [15]. Thus, DCB provides an opportunity to slow down or prevent secondary cell wall cellulose accumulation in a way that is the converse of the high cellulose accumulation that occurs via G-layer formation during tension wood production.

The aim of the present work was to investigate possible routes to the modification of willow cell wall structure and composition, which affect enzymatic saccharification. The use of DCB may help to establish a tool for the induction of useful phenotypes of value in lignocellulosic biofuel research. Increases in enzymatic saccharification yields achieved through changes in tree development could be independent of pretreatment and downstream processing methodologies (and the associated energy and environmental costs). Such knowledge may be used to further the development of sustainable, high-yielding, dedicated crops for the optimised production of biofuels to substitute for fossil-based liquid fuels and to mitigate greenhouse gas emissions from transport.

## Materials and methods

### Plant material, study site and experimental design

Cuttings of *Salix viminalis *× *Salix schwerinii *cultivar 'Olof' (20 cm in length and 1 to 1.5 cm in diameter) were grown in 12-L pots containing 10 L of growth media (one-third medium vermiculite, one-third sharp sand and one-third compost; John Innes no. 2 by volume) under glasshouse conditions of a 16-hour day (23°C) and an eight-hour night (18°C) for 91 days until harvested. Bamboo canes (2.7 m) were used to provide support. During the first week after planting, manual pruning of buds was applied so that each tree had only three stems to facilitate subsequent analysis of internal stem architecture.

The 26-day treatment experiment consisted of four treatments with six biological replicates per treatment. For the two inclined treatments (tension wood and DCB + tension wood), plant pots were positioned at a 45° angle to the floor. All inclined trees were tied to bamboo canes at regular intervals up the stem to prevent the normal gravitropic response at the apical meristem. It is presumed that tree ties also served to displace the majority of the load stress onto the bamboo canes in the inclined trees [16]. Each DCB-treated plant received a single application of 1 L of 58 μM DCB in 0.2% dimethyl sulfoxide (DMSO) solution, which applied 10 mg of DCB to the soil of the pot for each treated tree. These 24 trees were initially grown without treatment for 65 days before any treatment was applied, with further growth being allowed for a further 26 days before the trees were harvested. The 26-day treatment experimental design consisted of four rows of six trees. Initially, each tree received 200 mL of water daily, but this was increased to 400 mL daily after 50 days of growth.

To isolate the reaction wood components of the stems, a further six trees were grown: three inclined at 45° (as above), which were used for the isolation of tension and opposite wood, and three grown without treatment, which were used for the isolation of 'normal' wood. The growth conditions were similar to those described above, except for the induction of tension wood 17 days earlier, resulting in a tension wood treatment time of 43 days.

### Sampling and processing

**Figure 1:**
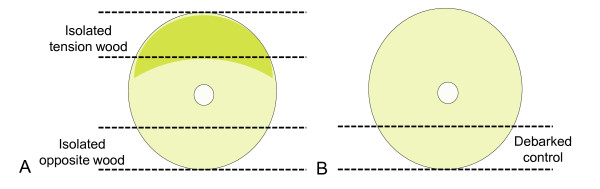
**Diagram of the transverse sections isolated to represent normal, tension and opposite wood fractions**. **(A) **Diagram of a transverse section of one of the three trees induced over 43 days by inclining. The two areas indicated by dashed lines represent the isolated tension wood and opposite wood fractions. **(B) **Diagram of a transverse section of one of the three control trees grown with the 43-day-treated trees. The single area indicated by the dashed line represents isolated fraction equivalent to reaction wood.

Only above-ground stem biomass was harvested, and the leaves were removed and discarded. A stem section (2 cm) was removed from the midpoint of each stem and fixed in

formaldehyde-acetic acid-alcohol solution (3.7% formaldehyde, 5% acetic acid and 47% alcohol) for later sectioning and microscopy. For the 26-day treatments, the remainder of the stem material for all the trees was processed for compositional analysis and enzymatic saccharification (see Enzymatic saccharification and Compositional analysis). For the 43-day treatments, all stem material from six trees was debarked and 2-cm longitudinal sections were cut (manually) from the entire length of the stem. The longitudinal sections were taken from the tension wood and opposite wood sides in the three tilted trees and from an equivalent position in the control trees (see Figure 1). All the harvested stem biomass was reduced separately to a particle size between 850 and 125 μm in accordance with National Renewable Energy Laboratory (NREL) Preparation of Samples for Compositional Analysis [17] using a Cutting Mill SM 2000 (Retsch, Haan, Germany).

### Sectioning, staining and microscopy

Transverse sections (15- to 20- μm thickness) of the midpoint stem samples from each of the six biological replicates for each of the 26-day treatment trees were cut using a Reichert sledge microtome Staining was performed using aqueous Safranin O and Chlorazol Black E [18], and permanent mounts on glass slides were made in DPX mounting medium

### Enzymatic saccharification

Enzymatic saccharification was performed as described previously [19] using a procedure in which the ground samples are incubated for seven days at a temperature of 50°C at pH 4.8. The exceptions were that the amount of sample added to the assay was normalised by dry matter (DM), 0.2 g, and the concentration of the cellulase enzyme mix was doubled to approximately 60 FPU/g DM cellulase (Celluclast 1.5 L; Novozymes, Bafsvaerd, Demark) and 64 pNPGU/g DM β-glucosidase (Novozyme 188; Novozymes, Bafsvaerd, Demark). The NREL protocol is designed to determine the maximum extent of digestion possible (via a saturating level of enzyme and extended incubation time). Doubling of enzyme concentration was used to further ensure that enzyme concentration was nonlimiting in the assay. Enzymatic saccharification was repeated in triplicate (with a duplicate substrate blank) for each of the six trees of the control and three 26-day treatments: (1) tension wood, (2) DCB and (3) tension wood-induced plus DCB. Enzymatic saccharification of the isolated reaction wood material from each of the three 43-day treatment trees was performed with a single enzyme reaction and single substrate blank, owing to the limited amounts of isolated material available. No specific pretreatment of the biomass was performed prior to enzymatic saccharification other than particle size reduction.

Enzymatic saccharification-released glucose is presented as a proportion of stem biomass DM and also back-calculated as a proportion of stem biomass glucan within each reaction with the caveat that the concentration of the cellulase mix was constant in each total mass of biomass but not according to the weight of glucan due to variations in the glucan content between the biomass types. However, as the concentration of cellulase enzymes was doubled, it was considered to be nonlimiting in the assay.

### Compositional analysis

Cell wall composition analysis for all biological replicates was performed in accordance with NREL Determination of Structural Carbohydrates and Lignin in Biomass [20] using material extracted using an ethanol-water mix in a Dionex Accelerated Solvent Extractor 300 (Dionex, Sunnyvale, California, USA) under conditions described in NREL Determination of Extractives in Biomass [21]. Unaccounted for mass is recognised here as 'Other' [4,22]. All sugar concentrations were determined using a Jasco Systems Intelligent HPLC (JASCO UK, Great Dunmow, Essex, UK) with a Bio-Rad Aminex HPX 87H column and refractive index detector (Bio-Rad Laboratories, Hemel Hempstead, Hertforshire, UK).

### Data and statistical analysis

We present three separate aspects of importance in defining biomass enzymatic saccharification potential: glucan content, released glucose (g^-1 ^glucan) and released glucose (g^-1 ^DM). The glucan content is the tree's absolute amount of cell wall glucan potentially available for fermentation into bioethanol expressed as a proportion of total biomass DM (including extractables and 'Other'). Released glucose (g^-1 ^glucan) is the proportion of glucose released from the available glucan within the biomass and so provides an indication to the accessibility of that glucan to depolymerisation enzymes. Hence, it is a measure of biomass recalcitrance to enzymatic saccharification. Released glucose (g^-1 ^DM) is a function of both the absolute glucan content and the accessibility of that glucan for each biological replicate.

Means and standard errors of the mean (mean ± SE) for each treatment were calculated from the six or three biological replicates. Analysis of variance (ANOVA) was performed for ten parameters using data from all 24 trees in the 26-day treatment experiment: total stem DM; percentages of glucan, xylan, arabinose, mannose, total lignin, 95% EtOH extractives and 'Other'; released glucose (g^-1 ^glucan); and released glucose (g^-1 ^DM). Tukey's Honestly Significant Difference (HSD) *post hoc *test [23] was performed for all parameters highlighted by ANOVA as significantly different, allowing pairwise comparisons between each treatment.

## Results and discussion

### Microscopic observations

**Figure 2:**
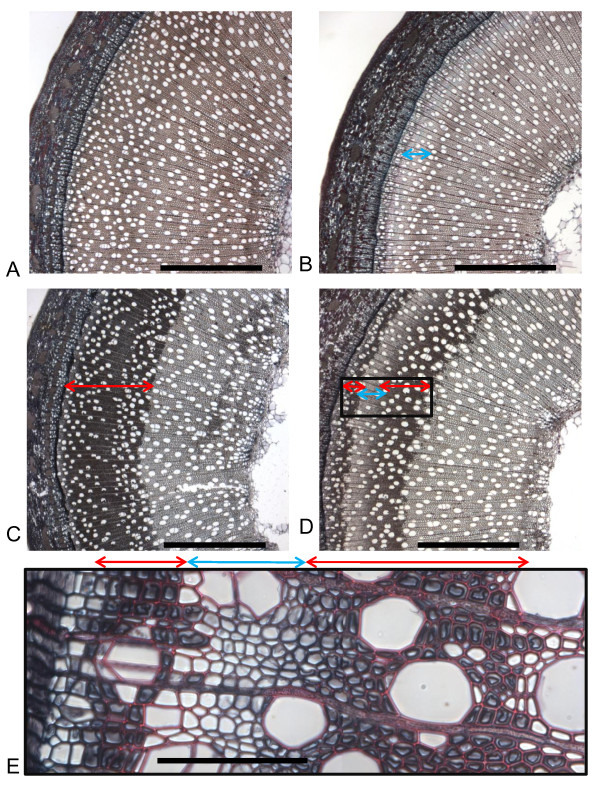
**Transverse sections of 26-day treatment groups**. Midpoint 20- μm transverse sections from the 26-day treatment groups stained in 1% Chlorazol Black E in methoxyethanol and 1% Safranin O (aqueous) of **(A) **control tree, **(B) **2,6-dichlorobenzonitrile (DCB)-treated tree, **(C) **tension wood-induced tree and **(D) **tension wood-induced, DCB-treated tree. Scale bar = 1 mm. **(E) **Tension wood-induced, DCB-treated tree. Scale bar = 100 μm. Tension wood- and DCB-affected regions are highlighted by vertical double arrows.

A distinctive layer of tension wood was formed along the entire length of the stem on only the upper side (negatively orientated to the vector of gravitational stimulus, regardless of horizontal bending of the stem) in all the tension wood induction trees. Successful induction of G fibres for each of the six 26-day tension wood induction trees was observed with G-layer formation in the secondary xylem fibre cells at the midpoint of every stem. Representative micrographs of the 26-day treatments are shown in Figures 2A through 2E. The formation of the G-layer within fibre cells occurred within a zone of between four to nine cells medial to the cambium. Safranin O staining also clearly highlighted that lignification of the secondary cell wall occurred only after the G-layers had already been assembled and after the apparent loss of the protoplast.

In the 26-day DCB-treated trees (Figure 2B), a band of fibre cells unstained by Safranin O was visible, in marked contrast to the control stems (Figure 2A). DCB plus tension wood-induced 26-day trees did initially produce G fibres in the same manner as 'standard' 26-day tension wood-induced trees without the DCB treatment before the DCB took effect (Figure 2D). This DCB effect arrested G-layer formation and also lignification, as revealed by Safranin O staining (Figure 2E). Both lignification and G-layer formation recovered sometime after the period of DCB effect, showing that the effect was transitory, presumably because of this being a concentration and/or depletion effect. Microscopic analysis indicated that lignification recovered before the recovery of G-layer formation. As tension wood formation can occur between 24 and 48 hours after a sufficient induction stimulus [24], a rough cell count comparison between the 26-day treatment tension wood-induced trees with and without DCB suggests that the DCB took around 10 days to begin to affect development at the cambium (which would be the midpoint of the stem after 91 days of growth). Using the same indicators, we estimated that the DCB effect lasted approximately 12 days. This information could be used to time DCB treatments and contribute to the construction of such secondary cell wall-specific experimental systems.

### Enzymatic saccharification and compositional analysis

The amount of released glucose (g^-1 ^DM) can be used in conjunction with biomass yield, required nutrient and water input and response, pest resistance, postharvest viability, plantation longevity and so forth as part of a complete ideotype for lignocellulosic biofuel assessment. By investigating 'native' biomass which had not undergone pretreatment, alteration of inherent cellulose accessibility was possible. However, it has yet to be established how changes in glucan content and released glucose (g^-1 ^glucan) of biomass will interact with any contemporaneous pretreatments and consequently the impacts on released glucose (g^-1 ^DM) and the energy and/or severity required to achieve optimal glucose (or other carbohydrate) yields.

**Figure 3:**
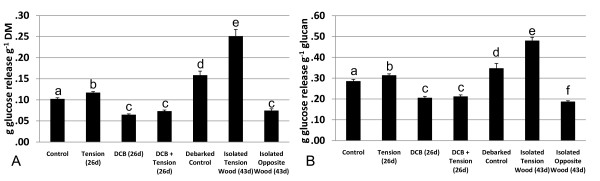
**Enzymatic saccharification**. Enzymatic saccharification of control, tension wood-induced, 2,6-dichlorobenzonitrile (DCB)-treated, and tension wood-induced, DCB-treated trees in the 26-day treatments group (26d, *n *= 6). Debarked control trees and isolated tension and opposite wood are also included for comparison in the 43-day treatment group (43d, *n *= 3). **(A) **g (grams) released glucose (g^-1 ^dry matter (DM)) and **B) **g released glucose (g^-1 ^glucan) Error bars = 1 standard error of the mean. Results of Tukey's Honestly Significant Difference (HSD) *post hoc *test (α = 0.05) are represented by a, b, c, d, e and f.

Control trees from the 26-day treatments had a mean glucan content of 35.8% DM (± 0.38) and a total lignin content of 26.5% DM (± 0.30). The most abundant hemicellulose monomer was xylose at 10.8% DM (± 0.25) (Table 1). For saccharification, control trees had 0.10 g (grams) released glucose g^-1 ^DM (± 0.0027) and 0.29 g released glucose g^-1 ^glucan (± 0.0078) (Figure 3). Although the composition was relatively stable between the six biological replicates of the 26-day control trees, it should be remembered that this composition is for juvenile trees grown in the glasshouse conditions of this pot trial.

**Table 1:** Stem biomass composition^a^

	95% EtOH extractives, % DM	**Glucan**, % DM	**Xylan**, % DM	Galactan, % DM	Arabinan, % DM	Mannan, % DM	ASL, % DM	AIL, % DM	Other, % DM
Control	8.3 (0.7)	35.8 (0.4)	10.8 (0.3)	3.0 (0.2)	2.7 (0.5)	2.0 (0.2)	4.3 (0.1)	22.3 (0.3)	10.9 (1.0)
Tension wood, 26 days	7.4 (0.4)	37.4 (1.0)	10.7 (0.5)	3.0 (0.1)	2.7 (0.4)	2.0 (0.2)	4.2 (0.2)	22.2 (0.5)	10.4 (1.4)
DCB, 26 days	9.2 (0.6)	31.4 (0.4)	9.8 (0.2)	3.0 (0.2)	3.4 (0.4)	2.0 (0.2)	4.4 (0.2)	24.1 (0.3)	12.6 (1.5)
DCB tension wood, 26 days	8.5 (0.5)	34.2 (0.8)	10.2 (0.3)	3.0 (0.2)	3.2 (0.4)	2.2 (0.4)	4.1 (0.1)	23.9 (0.5)	10.7 (1.5)
Debarked control	4.7 (0.2)	45.7 (0.3)	15.8 (1.0)	3.6 (0.6)	0.3 (0.5)	0.0	4.3 (0.10)	19.0 (0.5)	6.2 (2.6)
Isolated tension wood, 43 days	5.6 (0.3)	52.20 (1.8)	14.80 (0.8)	4.6 (0.5)	0.2 (0.2)	0.0	3.8 (0.1)	17.1 (0.3)	1.7 (2.1)
Isolated opposite wood, 43 days	8.7 (2.8)	39.8 (1.3)	16.3 (0.5)	3.1 (0.4)	0.0	0.0	4.3 (0.2)	18.6 (1.2)	9.1 (2.2)

^a^ASL, acid-soluble lignin; AIL, acid-insoluble lignin; DM, dry matter; DCB, 2,6-dichlorobenzonitrile. Data represent mean stem biomass composition with standard error of the mean shown in parentheses. For control, *n *= 6 for tension wood, DCB-treated, and DCB-treated, tension wood-induced trees in the 26-day treatment group; for debarked control, *n *= 3 for isolated tension wood and isolated opposite wood 43-day treatment group. Unaccounted for mass is recognised here as Other.

**Figure 4:**
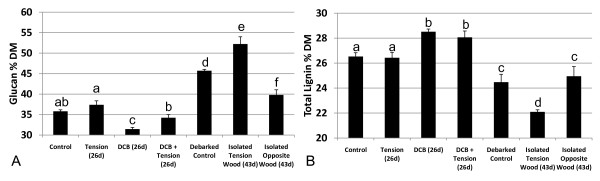
**Compositional analysis**. Composition of control, tension wood-induced, 2,6-dichlorobenzonitrile (DCB)-treated, and tension wood-induced, DCB-treated trees in the 26-day treatment group (*n *= 6). Debarked control trees and isolated tension and opposite wood are also included for comparison in the 43-day treatment group (*n *= 3). **(A) **Glucan content expressed as the percentage of dry matter (% DM). **(B) **Total lignin content expressed as % DM. Error bars = 1 standard error of the mean. Results of Tukey's Honestly Significant Difference (HSD) *post hoc *test (α = 0.05) are represented by a, b, c, d, e and f.

Significant variation was found within the glucan and total lignin contents and in the released glucose (g^-1 ^DM) and released glucose (g^-1 ^glucan) saccharification results between the different treatments. No significant variation from control was obtained between any of the 26-day trees for total stem DM or for the percentage of extractables, xylose, galactose, arabinose or mannose. Tukey's HSD *post hoc *multiple comparisons test was performed to verify the significant difference between treatments, and the results (α = 0.05) are indicated by letters in Figures 3 and 4.

### Effect of tension wood induction on composition and saccharification

Tension wood induction significantly increased both the released glucose (g^-1 ^DM) and released glucose (g^-1 ^glucan) yields of the whole tree after 26-day induction treatments compared with the controls by 15% and 10%, respectively. However, the 26-day tension wood induction treatments did not significantly affect any of the assayed elements of total stem composition compared with control trees (Figures 3 and 4 and Table 1). The increased glucan accessibility in the absence of significantly different levels of any cell wall polymers suggests that G-layers, clearly visible by microscopy (Figure 2C), contain more accessible glucan. The 26-day treatment, although long enough to influence glucose release yields, was a comparatively short portion of the 91 days of total growth. More prolonged or acute tension wood induction has been shown to produce high-glucan, low-lignin phenotypes [18,25,26].

Quantitatively assaying the proportion of tension wood within an induced tree remains difficult. However, subsequent compositional analysis and enzymatic saccharification of isolation of G fibre-rich tension wood from the 43-day treatment trees did indeed reveal not only that tension wood contains increased levels of glucan compared with the debarked controls (14% increase in glucan content) but also that the accessibility of this glucan is increased (namely, a 38% increase in released glucose g^-1 ^glucan) (Figures 3B and 4A). Interestingly, the increase in accessibility in isolated tension wood is significantly less obvious when assessing the whole tree, and the increase in glucan content is entirely lost (namely, the 26-day treatment results). Analysis of the isolated opposite wood from the 43-day treatments also sheds light on this disparity as, conversely, it has a lower glucan content than debarked control stems (13% decrease in glucan content) but also less accessible glucan (a 46% decrease in released glucose g^-1 ^glucan). Hence, we believe that the less favourable composition and accessibility of opposite wood acts to severely counteract the beneficial effects on saccharification yields for the tension wood material. The degree of asymmetric growth and visible tension wood, and hence the ratio of the tension wood to opposite wood fraction, is of significant importance when looking at effects on saccharification yields at the whole-tree level.

Debarked control trees were used as a comparison as isolated reaction wood would not be composed of any bark. By comparing the control trees from the 26-day treatments and the debarked control trees from the 43-day treatments, we can see the impact of bark on our analysis. Debarking results in biomass with a decrease in lignin content, an increase in glucan content and its accessibility and, consequently, an increase in released glucose (g^-1 ^DM) by 55% (Figure 3A).

### The effect of DCB on composition and saccharification

The absence of alteration in the aggregated cell wall polymer contents between the 26-day tension wood-induced whole trees and control trees is in marked contrast to DCB-treated trees, in which significantly reduced glucan and increased lignin levels were found compared with control trees (Table 1 and Figure 4). When released glucose yield was corrected for the reduced glucan present in DCB-treated trees, there was still a considerable reduction of 37% in released glucose (g^-1 ^glucan) compared with control trees (Figure 3B). These compositional changes show not only that DCB treatment successfully resulted in inhibition of secondary cell wall cellulose accumulation (reduced glucan) but also that the glucan within that cell wall is also more recalcitrant to enzymatic saccharification. Plants treated with 0.2% DMSO as controls for the DCB treatments showed no variation in growth from control plants, other than slight discolouration at the leaf edges. It should be noted that the substantial effects of the DCB and the tension wood induction treatments on cell wall composition did not affect biomass yields over the 91 days of growth.

### The effect of tension wood induction and DCB treatment on composition and saccharification

The induction of tension wood formation in DCB-treated, 26-day trees led to a glucan content that was significantly increased, restoring it to levels not significantly different from those of control trees (Table 1 and Figure 4). Lignin levels remained higher than in control trees at levels not significantly different from the elevated levels found in DCB-treated trees without tension wood induction. The released glucose (g^-1 ^glucan) also remained the same in DCB plus tension wood-induced trees as in DCB-treated trees without tension wood induction, indicating that even though the glucan levels had been increased via the tension wood induction, the accessibility of that glucan was not restored to control tree levels (Figure 4A). It has been proposed that the available glucose pool, instead of the cellulose synthesis machinery itself, is the limiting factor in cell wall cellulose synthesis [27]. As DCB-treated trees had reduced glucan content, the available glucose pool, of which starch is the major nonpermanent glucose storage molecule, might be increased, so it is interesting that tension wood induction led to a much greater increase in glucan levels in DCB-treated trees compared with control trees. The primary role of the glucose pool in tension wood formation is also corroborated by large increases in transcript levels of sucrose synthases SUS1 and SUS2 during tension wood formation [27,28]. It can be hypothesised that this allows the necessary increase in allocation of carbon to the site of tension wood formation by mediating increased glucose partitioning from starch to sucrose (the trees' primary carbon transport molecule). The inability of tension wood induction to significantly increase whole-tree glucan levels due to a depleted and/or limited accessible glucose pool in control trees could be attributable to their extremely rapid growth. This may give rise to a photoassimilate limitation of growth, owing to the controlled conditions and very high nutrient and water inputs compared with field conditions.

### The role of lignin

Interestingly, the role of lignin in the accessibility of the cell wall to degrading enzymes seems dissimilar between willow from the tension wood inductions and DCB treatments. In DCB-treated trees with induced tension wood, despite glucan levels' being restored to those of control trees, lignin levels were also much higher than those in control trees. In this case, cell wall glucan accessibility was greatly reduced, suggesting that lignin levels may influence cell wall recalcitrance to enzymatic saccharification.

In contrast, in tension wood-induced trees, the lignin content was not significantly different from that of controls at a whole-tree level (26-day inductions), but released glucose (g^-1 ^glucan) yields were significantly elevated, suggesting that overall lignin levels do not play a strong role in influencing glucan accessibility (Figures 3 and 4 and Table 1). This is further supported by the isolated reaction wood fractions (43-day inductions), where opposite wood had the same lignin content as normal wood controls, yet had significantly reduced glucan accessibility. These results suggest that in non-pretreated biomass, lignin is not always a significant factor in glucan accessibility to enzymatic saccharification.

Although tension wood induction did not produce a whole-tree, low-lignin phenotype, this is believed to be due to the short period of induction. Isolation of the tension wood did indeed show this fraction to have reduced lignin. Much of the current research, with the goal of developing high sugar-yielding phenotypes for biofuels, has focused on interrupting lignin biosynthesis pathways [29-33]. The shifts in transcription levels during tension wood formation have been investigated [27] and seem to provide insight into potentially fruitful targets for modifying aspects such as lignin biosynthesis to produce low-lignin phenotypes. As much of the regulation of lignin synthesis and the physiological role of lignin within the cell wall are still unclear, examination of extreme variant phenotypes, representing natural metabolic plasticity, present a profitable research opportunity whilst not compromising plant integrity in commercial application.

### The impact of cell wall modification at a developmental level

The aim of this work was to modify cell wall composition and enzymatic saccharification at the whole-stem level. This was successfully achieved in the 26-day treatments whilst maintaining above-ground biomass yields through the induction of G fibres and the interruption of cellulose synthesis. We have revealed disparate effects on the enzymatic saccharification of willow biomass by using two different treatments affecting the cell walls in a different manner. DCB effectively decreased secondary cell wall glucan accessibility and content. Tension wood induction instigated the assembly of a unique cell wall layer which increased overall cellulose content and accessibility. These findings further demonstrate that optimal selection traits in biomass feedstocks for lignocellulosic biofuel need to include not only biomass yield but also biomass composition and cell wall accessibility to enzymatic digestion.

The present study used a single cultivar 'Olof'; however, the *Salix *genus contains vast genetic and phenotypic diversity [34] which has not been investigated in detail for cell wall accessibility to enzymatic digestion. Further work should assess the occurrence and induction characteristics of tension wood across this broad natural variation to expand the potential understanding of cell wall construction, functionality and conversion. In this study, extreme phenotypes in cell wall accessibility were induced. It has yet to be established whether the roles suggested here regarding how glucan and lignin content affect released glucose yields are maintained in naturally occurring phenotypes and how tension wood induction approaches may affect biomass yields in field-grown material. Both of these aspects must be explored before these findings can be applied to the breeding of biofuel willows.

## Conclusions

Tension wood induction in the willow cultivar 'Olof' led to significant increases in glucose yields via enzymatic saccharification in comparison with control trees. The results demonstrate that induction of naturally occurring phenotypes in biomass to achieve significantly enhanced glucose yields provides one avenue by which to minimise the energy and environmental impacts of lignocellulosic pretreatment technologies.

## Competing interests

The authors declare that they have no competing interests.

## Authors' contributions

NJBB conceived the study; participated in the biomass cultivation, processing, compositional and saccharification assays; performed the statistical analysis; and drafted the manuscript. FEP participated in the design of the study and in biomass processing. MJR participated in biomass cultivation and in compositional and saccharification assays. AK and RJM also conceived and participated in the design and coordination of the study. All the authors contributed to, read and approved the final manuscript.
